# The epigenetic regulator G9a attenuates stress-induced resistance and metabolic transcriptional programs across different stressors and species

**DOI:** 10.1186/s12915-021-01025-0

**Published:** 2021-05-24

**Authors:** Human Riahi, Michaela Fenckova, Kayla J. Goruk, Annette Schenck, Jamie M. Kramer

**Affiliations:** 1 Department of Human Genetics, Donders Institute for Brain, Cognition and Behaviour, Radboud University Medical Center, Nijmegen, The Netherlands; 2 Department of Biochemistry and Molecular Biology, Dalhousie University, Halifax, Nova Scotia Canada

**Keywords:** Stress response, Resistance, Tolerance, G9a, Transcription, Drosophila, Mammalian cells, Metabolism

## Abstract

**Background:**

Resistance and tolerance are two coexisting defense strategies for fighting infections. Resistance is mediated by signaling pathways that induce transcriptional activation of resistance factors that directly eliminate the pathogen. Tolerance refers to adaptations that limit the health impact of a given pathogen burden, without targeting the infectious agent. The key players governing immune tolerance are largely unknown. In *Drosophila*, the histone H3 lysine 9 (H3K9) methyltransferase G9a was shown to mediate tolerance to virus infection and oxidative stress (OS), suggesting that abiotic stresses like OS may also evoke tolerance mechanisms. In response to both virus and OS, stress resistance genes were overinduced in *Drosophila* G9a mutants, suggesting an intact but overactive stress response. We recently demonstrated that G9a promotes tolerance to OS by maintaining metabolic homeostasis and safeguarding energy availability, but it remained unclear if this mechanism also applies to viral infection, or is conserved in other species and stress responses. To address these questions, we analyzed publicly available datasets from *Drosophila*, mouse, and human in which global gene expression levels were measured in G9a-depleted conditions and controls at different time points upon stress exposure.

**Results:**

In all investigated datasets, G9a attenuates the transcriptional stress responses that confer resistance against the encountered stressor. Comparative analysis of conserved G9a-dependent stress response genes suggests that G9a is an intimate part of the design principles of stress resistance, buffering the induction of promiscuous stress signaling pathways and stress-specific resistance factors. Importantly, we find stress-dependent downregulation of metabolic genes to also be dependent on G9a across all of the tested datasets.

**Conclusions:**

These results suggest that G9a sets the balance between activation of resistance genes and maintaining metabolic homeostasis, thereby ensuring optimal organismal performance during exposure to diverse types of stress across different species. We therefore propose G9a as a potentially conserved master regulator underlying the widely important, yet poorly understood, concept of stress tolerance.

**Supplementary Information:**

The online version contains supplementary material available at 10.1186/s12915-021-01025-0.

## Background

Multicellular organisms have evolved mechanisms to defend themselves against harmful agents in their environment [1]. These can be biotic factors like infectious microorganisms and parasites, or abiotic factors such as oxidative stress (OS) and hypoxia. Organisms use two complementary strategies to protect themselves against infectious microorganisms: resistance and tolerance [2–4]. In immune responses, resistance refers to mechanisms that are designed to eliminate the microbe from the host. Bacteria, for example, can be killed by expression of antimicrobial peptides, and invading pathogens can be encapsulated or phagocytosed by immune cells to eliminate infections. In contrast, tolerance refers to mechanisms that mitigate the damage caused by infection and the associated immune response of the host [2–4].

The host immune response is associated with systemic metabolic remodeling. In *Drosophila*, infection by a parasitoid wasp or bacteria causes glycogen breakdown and increased circulating glucose [3, 5, 6]. This increased glucose demand is thought to serve the increased energy needs of activated immune cells [6, 7]. In humans, the response to severe infection also involves a metabolic shift in leukocytes that is characterized by reduced oxidative phosphorylation, coupled with increased aerobic glycolysis, a metabolic state known as the Warburg effect. Oxidative phosphorylation may be too slow to meet the rapid energy needs of activated immune cells. Aerobic glycolysis, while less efficient, produces ATP faster and also prevents further accumulation of ROS, which is produced primarily during mitochondrial oxidative phosphorylation [8]. Aerobic glycolysis can also supply crucial intermediates that are used as building blocks for the synthesis of new cellular materials, like lipids, which may be required for vesicle formation during phagocytosis of pathogens [9]. Defects in immunometabolism are associated with sepsis and immune paralysis [10, 11]. However, while metabolic remodeling is essential for the immune response, it can also be harmful to the host organism because cell resources are diverted away from other physiological processes and can become limiting [3, 5, 12]. Therefore, attenuation of the immune response and the associated metabolic remodeling has been proposed as an important mechanism in immune tolerance [5, 12]. In addition, exciting recent work has shown that tolerance can be triggered by preventing inhibition of endogenous glucose production via liver gluconeogenesis or by inducing a hypometabolic state at low temperature [13, 14].

The regulatory factors driving tolerance are still poorly understood. One exception is the epigenetic regulator G9a, which was shown to be required for tolerance to virus infection in *Drosophila melanogaster* [15]. G9a mutants die faster than controls after exposure to the virus but maintain a normal viral load [15]. G9a is a histone methyltransferase that mediates dimethylation of histone H3 on lysine 9 (H3K9me2), which is an epigenetic mark that has been associated with repressed transcription [16]. In response to virus infection, the JAK/STAT pathway is activated, inducing key transcriptional targets involved in anti-viral defense. Through its role as an epigenetic repressor, G9a limits the induction of JAK/STAT-induced immune response genes. Overactivation of JAK/STAT signaling is harmful, suggesting that G9a promotes viral tolerance in part by preventing an overactive immune response [15].

G9a is also involved in preventing overactivation of resistance mechanisms against reactive oxygen species (ROS) in *Drosophila* [17]. ROS is eliminated by the OS response, a transcriptional program that upregulates resistance factors including superoxide dismutase and catalase, which together can convert harmful oxygen radicals into water. G9a mutants die faster than controls when exposed to OS and show overactivation of key OS resistance genes. G9a mutants ultimately die during OS exposure, not due to sustained oxidative damage, but due to a lack of available energy [17]. Therefore, in the *Drosophila* OS response, G9a promotes stress tolerance by maintaining the balance between activation of resistance genes and maintenance of metabolic homeostasis [17]. While *Drosophila* G9a does regulate tolerance to both virus and OS, it is unclear whether viral tolerance is linked to metabolic dysregulation.

G9a has also been implicated in biotic and abiotic stress responses in mammals. Regulation of interferon-mediated immune response in mouse embryonic fibroblasts (MEFs) was found to depend on G9a-mediated H3K9me2 [18]. The induction of interferon response genes is much higher in MEFs that are depleted of G9a, suggesting that G9a is responsible for buffering the mouse immune response [18], similar to what was observed during the *Drosophila* virus and OS response [15, 17]. G9a is also required for hypoxia response in human MCF-7 breast cancer cells and in mouse embryonic stem (ES) cells and was shown to buffer hypoxia-induced genes [19–21].

Taken together, these studies suggest that G9a might be a key factor in preventing overactivation of stress response genes. It is unclear, however, if mammalian G9a functions to promote stress tolerance and energy homeostasis, as observed in *Drosophila*. Here, we integrated publicly available *Drosophila* [15, 17] and mammalian [18, 19, 21] transcriptome datasets to determine whether G9a, in different species under different types of stress, drives a universal transcriptional core program that can shed light on the mechanisms underlying stress tolerance. Across the investigated datasets, we observe a striking conservation of G9a-dependent stress-responsive genes and biological processes. Our results suggest G9a as a conserved universal regulator of stress tolerance that balances the activation of stress resistance genes with the need to maintain metabolic homeostasis.

## Results

### G9a safeguards transcriptional homeostasis in different species under different types of stress

**Fig. 1:**
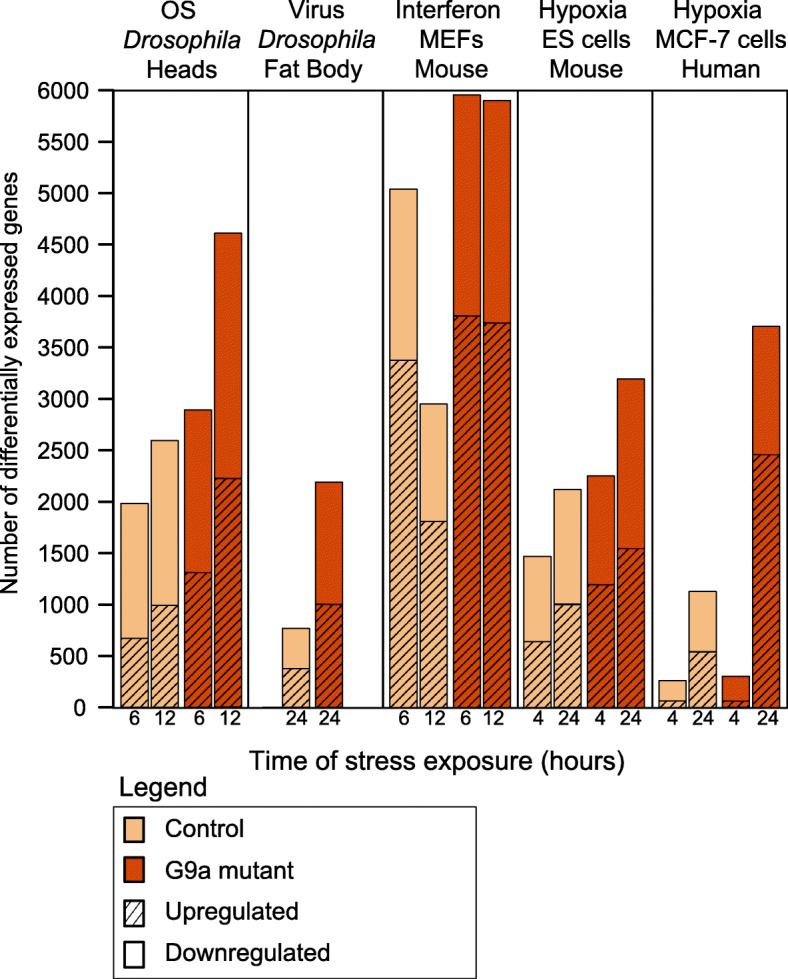
Stress-induced gene expression changes are increased in G9a-depleted conditions. Bar graph showing the number of differentially expressed genes identified in pairwise comparisons of mRNA levels before and after stress exposure in G9a-depleted conditions (orange) and controls (beige). Differentially expressed genes were defined by an adjusted *p* value < 0.05, and a fold change > 1.2 up (normal fill) or down (striped)

To investigate shared G9a-dependent transcriptional programs across stressors and species, we analyzed published G9a stressor datasets that provided transcriptome data from G9a-depleted systems and controls in non-induced conditions and upon stress exposure, at one or two time points past stress induction. We identified 5 such datasets: (1) adult *Drosophila G9a* mutants exposed to paraquat (oxidative stress *Drosophila*) [17], (2) adult *Drosophila G9a* mutants exposed to an RNA virus (virus *Drosophila*) [15], (3) G9a knockout MEFs exposed to Poly I:C immune stimulation (interferon MEF) [18], (4) G9a knockout mouse ES cells exposed to hypoxia (hypoxia ES) [21], and (5) a dataset collected upon pharmacological inhibition of G9a in human MCF-7 breast cancer cells exposed to hypoxia (hypoxia MCF-7) [19]. Collectively, these five transcriptome datasets represent 3 different organisms (fly, mouse, and human) and three general types of stress: oxidative, immune, and hypoxic stress. Within each dataset, we identified stress-responsive genes, which were defined as any gene showing a significant change in expression after exposure to stress *(p-*adj < 0.05, fold change > 1.2) (Table 1). Consistently, we observed a higher number of stress-responsive genes in G9a-depleted conditions compared to controls (Fig. 1). To determine if G9a might have a role in regulating the expression of these stress-responsive genes, we tested their differential expression in G9a-depleted conditions versus controls following exposure to stress. Across all datasets, we found that an average of > 40% of stress-responsive genes were dependent on G9a (Table 1), revealing a major contribution of G9a to stress-induced transcriptional programs.

**Table 1:** Identification of G9a-dependent stress-responsive genes

Dataset short name	Organism	Tissue or cell line	Type of stress	Exposure Time (h)	Stress-responsive genes^**1**^	G9a-dependent stress-responsive genes^**2**^	Percentage of stress-responsive genes dependent on G9a
Oxidative stress *Drosophila*	*Dm*	Whole head	Oxidative stress	6, 12	5737	2243	39%
Virus *Drosophila*	*Dm*	Fat body	Viral infection	24	2560	1290	50%
Interferon MEF	*Mm*	Embryonic fibroblasts	Interferon	6, 12	7311	2130	29%
Hypoxia ES	*Mm*	Embryonic stem cells	Hypoxia	4, 24	4165	1365	32%
Hypoxia MCF-7	*Hs*	MCF-7 cells	Hypoxia	4, 24	3757	1994	53%

*Dm - Drosophila melanogaster*, *Mm - Mus musculus, Hs - Homo sapiens*

^1^Stress-responsive genes are defined as a having significantly different expression levels (*p*-adj < 0.05, fold change > 1.2) in response to stress in controls or in G9a depleted conditions

^2^Stress-responsive genes that also have significantly different expression in G9a-depleted conditions compared to controls (*p*-adj < 0.05, fold change > 1.2)

**Fig. 2:**
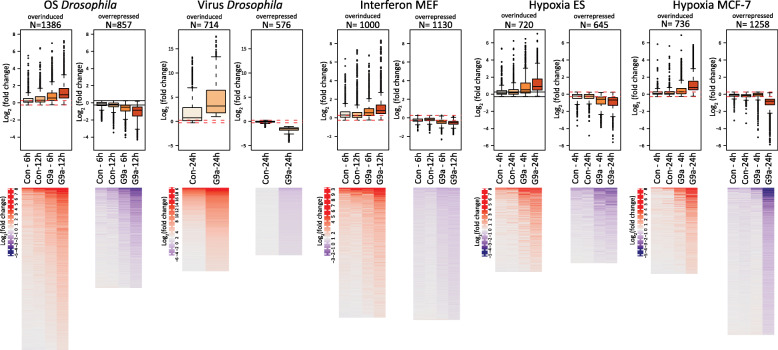
G9a attenuates stress-induced gene expression changes in different species under different types of stress. Boxplots and heat maps showing log2 fold changes of G9a-dependent stress-responsive genes across all five published datasets. Stress-dependent gene expression changes tend to be increased in magnitude in G9a-depleted conditions, being either overinduced or overrepressed after stress exposure. The numerical data depicted in this figure can be found in Table S1

Next, we examined the effect of G9a on the magnitude and direction of dysregulated stress-responsive genes. G9a-dependent stress-responsive genes generally showed an exaggerated response. Genes that were induced in controls, were more induced in G9a-depleted conditions, while genes that were repressed in response to stress in controls were more repressed in G9a-depleted systems. We refer to these groups as overinduced and overrepressed genes, respectively (Fig. 2, Table S1).

Taken together, our analysis demonstrates that G9a buffers stress-induced gene expression changes and maintains transcriptional homeostasis in different species under different types of stress.

### G9a regulates shared biological processes in different species under different types of stress

**Fig. 3:**
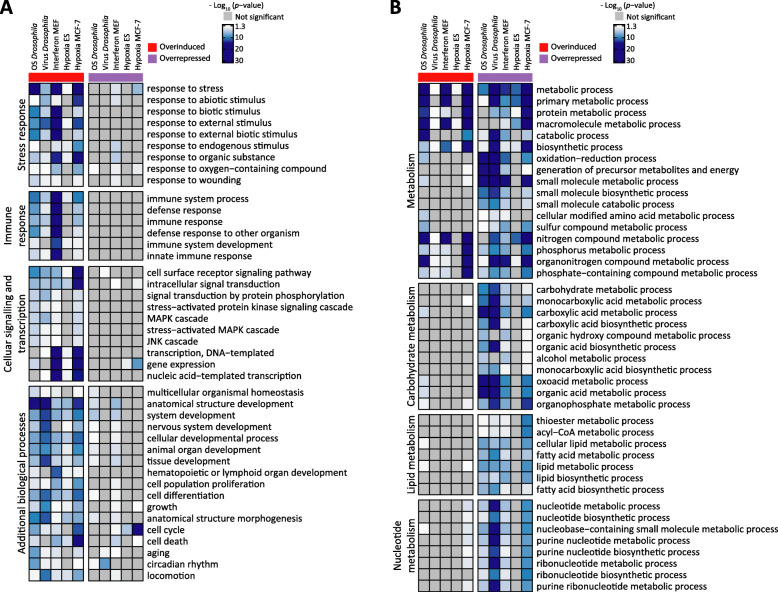
Stress response genes are overinduced and metabolic genes are overrepressed in G9a-depleted conditions. **a**, **b** Heatmaps showing *p* values for GO enrichment highlighted in light to dark blue. Not significant terms are highlighted gray. Columns show overinduced (column 1–5, annotated in red) and overrepressed (column 6–10, annotated in purple) gene groups from each dataset. Row colors indicate -log10 of *p* value (false discovery rate) for enrichment of the indicated GO terms in each dataset. Here we show all selected common GO terms subdivided into functional groups of **a** stress-related terms: stress response, immune response, cellular signaling and transcription, and additional biological processes; and **b** metabolic terms: metabolism, carbohydrate metabolism, lipid metabolism, and nucleotide metabolism

To identify the cellular pathways and functions that are under control of G9a during stress responses, we applied gene ontology (GO) analysis to the G9a-dependent overinduced and overrepressed gene sets. To provide a comparative overview across the 5 datasets, we constructed GO heatmaps displaying *p-*value significance for GO terms that are enriched in at least four of the five datasets (Fig. 3). Among G9a-dependent overinduced genes we observed enriched biological processes related to stress responses, immune responses (also a type of stress response), cellular signaling, and transcription. These themes were not generally enriched among G9a-dependent overrepressed genes (Fig. 3a). This demonstrates that G9a attenuates the induction of stress response genes in different species under different types of stress.

Both G9a-dependent overinduced and overrepressed genes are enriched for GO terms related to metabolism. Of these, terms related to small molecule metabolism, carbohydrate metabolism, lipid metabolism, and nucleotide metabolism show preferential enrichment among G9a-dependent overrepressed genes (Fig. 3b). These data suggest a remarkable conservation of G9a-dependent regulation of biological processes in the different analyzed stressors and species.

### G9a regulates stress-specific genes and common stress response genes

**Fig. 4:**
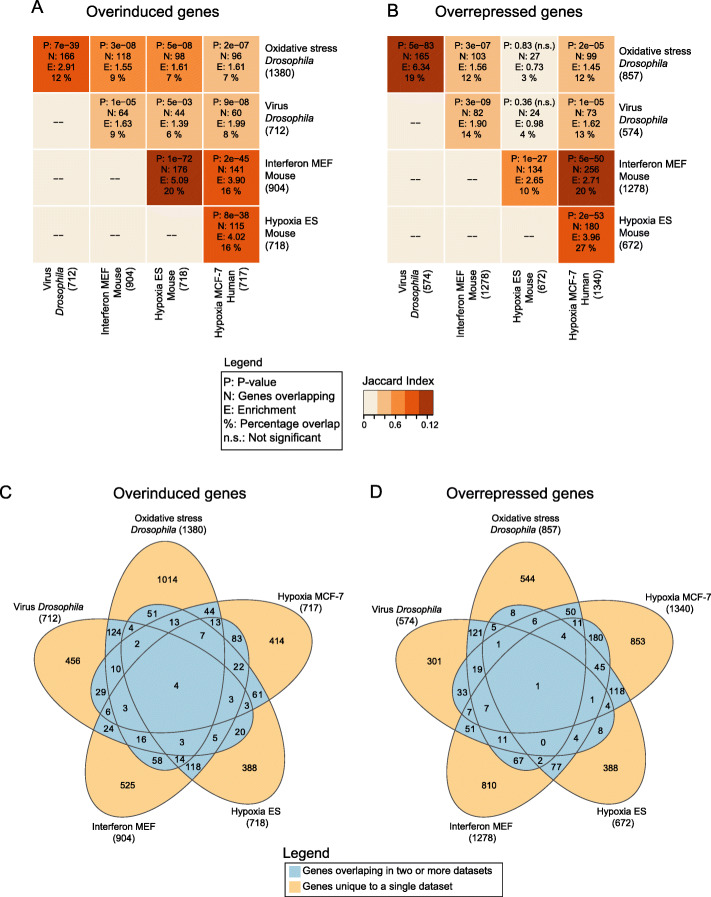
Identification of orthologous genes that are commonly regulated by G9a in different species under different types of stress. **a**, **b** Heatmaps indicating pairwise overlap of genes between the five datasets for the **a** overinduced and **b** overrepressed gene groups. The total number of fly genes, or fly orthologs of mouse and human genes, is indicated below the dataset name. Overlap statistics on each tile connecting two datasets include hypergeometric *p* value for the enrichment, number of overlapping genes, fold enrichment, and percentage of overlap. The similarity between two datasets is represented by the Jaccard index as a color gradient in the tile. **c**, **d** Venn diagram showing overlaps between datasets for the **c** overinduced and **d** overrepressed genes groups. Total number of fly genes, or fly orthologs of mouse and human genes, is indicated below the dataset name. Overlap between two or more groups is highlighted in blue and unique genes in each dataset are indicated in beige

Next, we further zoomed into the data and investigated conservation of G9a-dependent overinduced and overrepressed responses at the gene level. Pairwise comparisons between each dataset revealed a significant overlap in specific genes between most datasets for both overinduced (Fig. 4a) and overrepressed (Fig. 4b) gene groups (*p* < 0.05 for 18 of 20 comparisons, Jaccard Index). The most significant degrees of overlap were found between datasets derived from the same species (mouse and fly) or the same type of stress (hypoxia), however significant overlap was also observed among all other pairs of overinduced gene-sets and most other pairs of overrepressed gene-sets (Fig. 4a-b). In total, there were 740 overinduced and 841 overrepressed genes that overlap in two or more datasets (Fig. 4c-d, highlighted in blue), while a total of 2797 overinduced genes and 2895 overrepressed genes were unique to single datasets (Fig. 4c, d, highlighted in beige). The complete list of unique and overlapping genes is provided in Table S2.

**Fig. 5:**
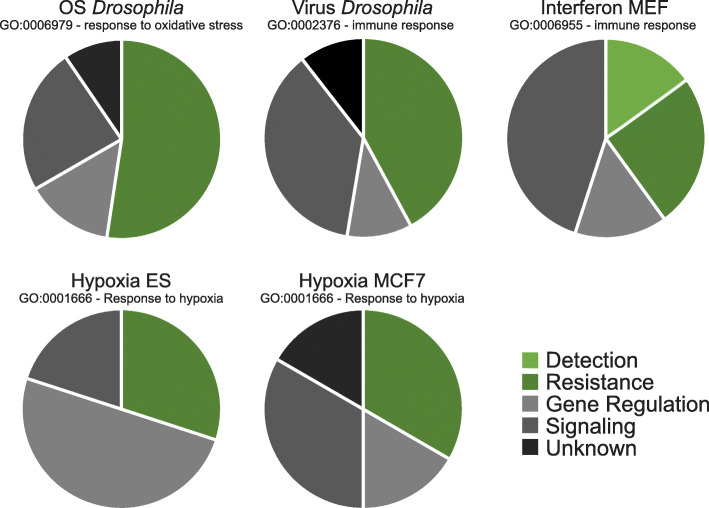
G9a buffers the activation of stress-specific resistance genes. We selected genes that were uniquely overinduced in only one of the five G9a-depleted datasets and were annotated with the specific GO terms “response to oxidative stress,” “immune response,” and “response to hypoxia.” Based on published evidence, we classified the function of each gene in the stress response in the following categories: detection (proteins that are involved in sensing the stress), resistance (proteins are directly involved in eliminating the stress), gene regulation (transcription factors, translation regulators, chromatin regulators, and cofactors that activate or repress a stress-responsive transcription factor), signaling (any molecule involved in a signal transduction pathway), and unknown (annotated in stress response based on a mutant phenotypes or transcriptional response without mechanistic understanding)

Attempting to understand the nature of gene sets that were dysregulated exclusively in a single versus multiple G9a stressor datasets, we first identified uniquely overinduced genes that were annotated with the specific GO terms “response to oxidative stress,” “immune response,” and “response to hypoxia.” We speculated that we would find stress-sensing receptors and downstream resistance factors among the unique targets, whereas components of promiscuous core signaling pathways would be shared. Interestingly, several known stress-specific resistance factors are found to be uniquely overinduced in the relevant transcriptome dataset (Table 2, Table S3). For example, in the *Drosophila* OS response, about half of the unique overinduced OS response genes are specifically involved in neutralizing oxygen radicals. The other half have roles in signaling and gene regulation that occurs in response to OS or were annotated based on a mutant phenotype without mechanistic understanding (Fig. 5, Table S3). The two immune datasets also showed about half of the uniquely overinduced immune response genes to be involved in immune resistance mechanisms such as encapsulation, activation of immune cells, and secretion of bactericides. About a third of unique overinduced hypoxia response genes were involved in hypoxia resistance, including factors involved in physiological adaptations to increase oxygen supply and removal of damaged or malignant hypoxic cells (Fig. 5, Table S3).

**Table 2:** Stress annotated genes that are uniquely overinduced in a single dataset

Dataset short name	GO term annotation	Genes
Oxidative stress *Drosophila*	Response to oxidative stress (GO:0006979)	PRX2540-1, INR, SPZ, P38A, CNC, CD, CAT, JAFRAC1, GSTE1, THOR, CG12896, WFS1, SIRUP, IRC, CG15547, PRX2540–2, MTF–1, P38C, LON, NAPRT, SCYL
Virus *Drosophila*	Immune response (GO:0002376)	UNC-45, THS, SPAS, P38B, NUB, LOLA, HH, DNR1, WNT4, VPS33B, TOTB, PVF3, PPO3, NT1, ECT4, DHC64C, CECC, CECB, ATTD
Interferon MEF	Immune response (GO:0006955)	LCK, TCIRG1, GATA3, KDM6B, TNFAIP3, STX8, RNF8, JAK2, ADAR, TLR2, D6WSU163E, OTUD5, TLR3, PLSCR1, MYD88, CASP4, CYLD, TLR4, HLX, DUSP10
Hypoxia ES	Response to hypoxia (GO:0001666)	SRF, APAF1, CAPN2, SCAP, BRIP1, HIGD1A, ADRB2, ECE1, PSME1, PSMB10
Hypoxia MCF-7	Response to hypoxia (GO:0001666)	NOS2, TGFB2, SOD2, VHL, UBQLN1, ADAM17, AK4, TGFBR2, SMAD3, ENDOG, RBPJ, RYR1

**Fig. 6:**
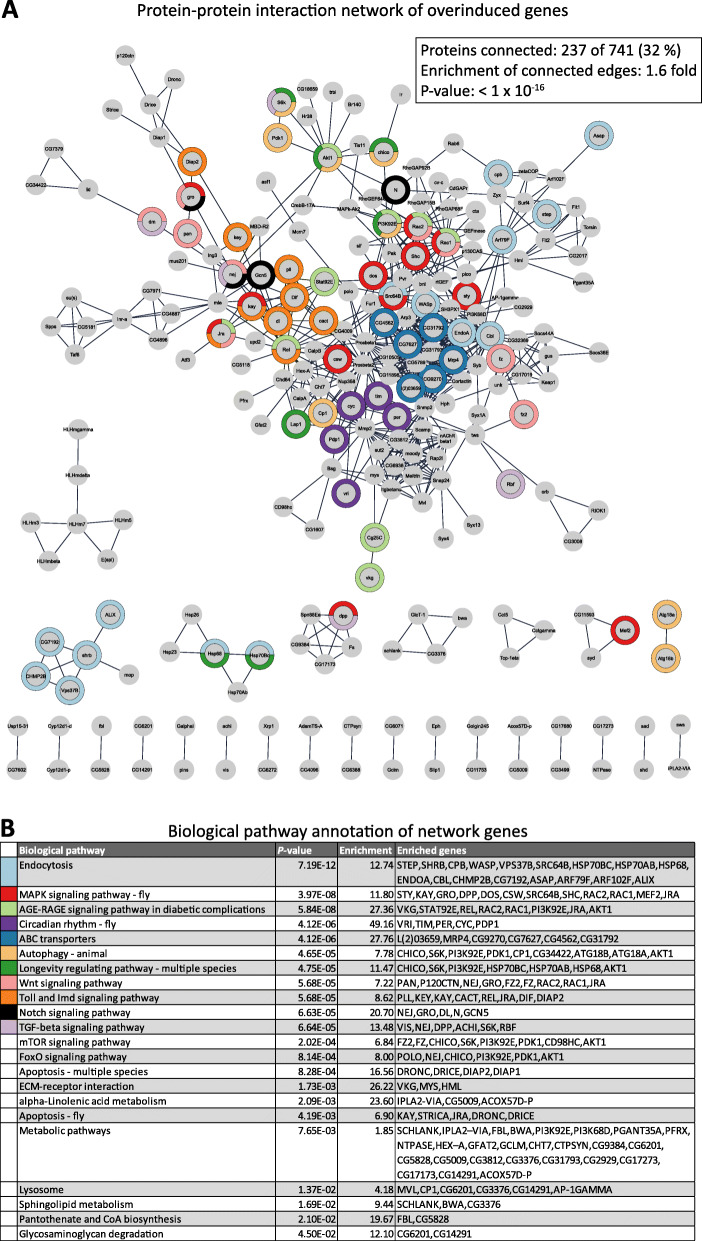
G9a attenuates a network of stress-activated genes in different species under different types of stress. **a** Protein interaction networks of overinduced overlap genes, as generated using the STRING app in Cytoscape. Of the 740 overinduced overlap genes, 237 are connected to at least one other gene in the group. The color-code indicates enriched KEGG terms associated with the networks, as indicated in **b**. **b** KEGG pathway enrichment of genes within the overinduced networks

Finally, we characterized conserved G9a-dependent stress-responsive genes that were dysregulated in multiple datasets. G9a-dependent overinduced genes that overlap in two or more datasets form a highly cohesive protein-protein interaction (PPI) network that is significantly enriched over background protein-protein connectivity (665 versus 420 randomly expected edges, *p* < 1*10^−16^, [22]) (Fig. 6). The network of overinduced genes is enriched for KEGG pathways related to major stress signaling pathways, such as Toll and Imd, AGE-RAGE, and MAPK signaling (Fig. 6). The conserved G9a-dependent stress-responsive genes are further enriched for KEGG terms at the intersection of stress signaling and metabolism/nutrient-sensing, including longevity-regulating pathways, mTOR signaling, endocytosis, autophagy, and lysosomes; as well as for genes annotated with the metabolic GO terms.

**Fig. 7:**
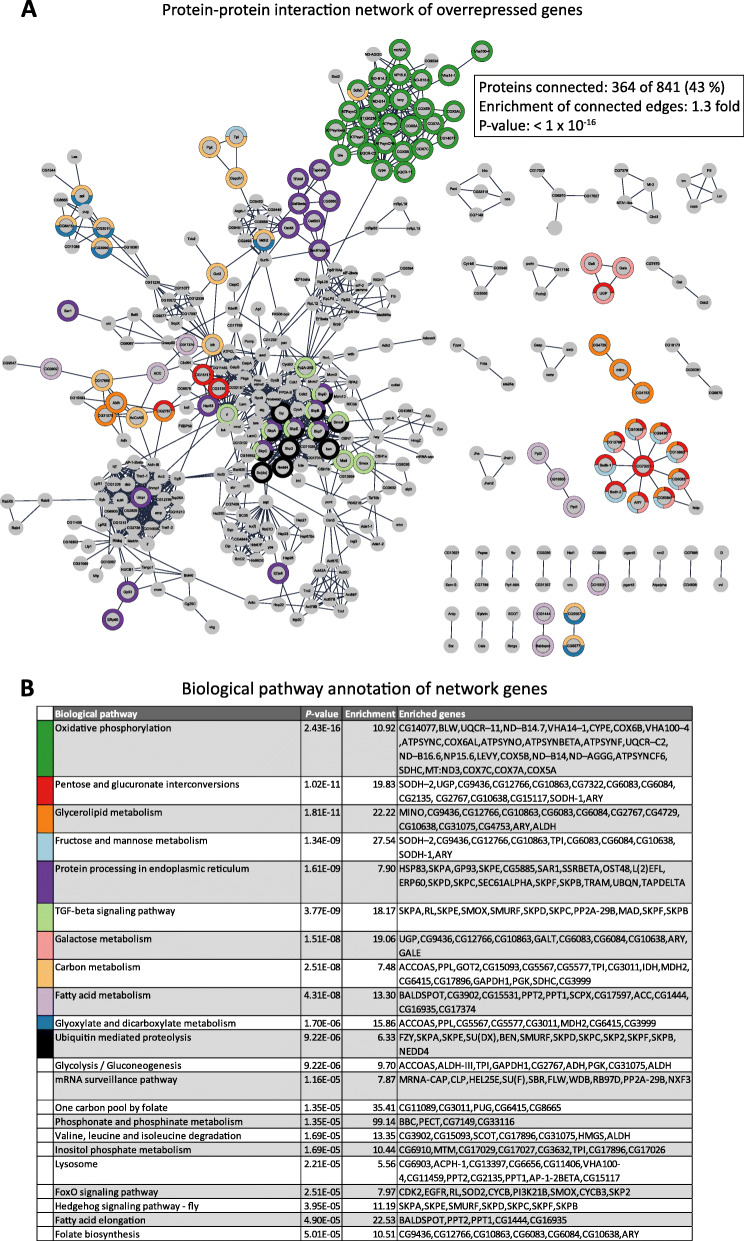
G9a attenuates a network of repressed genes in different species under different types of stress. **a** Protein interaction networks of overrepressed overlap genes, as generated using the STRING app in Cytoscape. Of the 841 overrepressed overlap genes, 364 are connected to at least one other gene in the group. The color-code indicates enriched KEGG terms associated with the networks, as indicated in **b**. **b** KEGG pathway enrichment of genes within the overrepressed networks

G9a-dependent overrepressed genes also form a PPI network with highly enriched connectivity (1270 edges versus 994 randomly expected, *p* < 1*10^−16^, [22]) (Fig. 7). Genes in this network are enriched for KEGG pathways related to energy metabolism, such as oxidative phosphorylation, sugar metabolism, and fatty acid metabolism, again underscoring the importance of G9a in the regulation of energy metabolism during various stress responses in different species under different types of stress.

## Discussion

In this study, we integrated five transcriptome datasets to investigate the conserved role of the epigenetic regulator G9a in the transcriptional response to different types of stress in different species and systems. Our results suggest that G9a is an intimate part of the design principle of stress resistance, buffering the inductions of stress-specific receptors, pleiotropic core stress signaling pathways, and stress-specific effectors. In addition to buffering the activated stress resistance program, G9a is also required across all five datasets for maintaining homeostasis of metabolic gene expression. Balancing the activation of cellular stress resistance programs with metabolic needs may be a fundamental mechanism underlying the poorly understood concept of disease tolerance. We therefore propose that G9a may be a universally conserved regulator of stress tolerance that sets the balance between stress resistance and metabolic homeostasis.

### G9a attenuates stress-induced transcription of the stress defense response

In all five G9a stressor datasets, we consistently observed transcriptional overinduction of stress response genes. Genes that were overinduced in multiple datasets were enriched for pleiotropic core components of known stress, immune, and metabolic signaling pathways, including Toll and Imd, AGE-RAGE, and MAPK signaling (Fig. 6).

Also of interest are the overinduced G9a targets that were specifically dysregulated in a single stress dataset. These encompassed key factors that are required to defend against the specific stressor to which the specific system had been exposed, as well as signaling molecules and transcription factors that are stress-specific, but also promiscuous ones including some that act in the conserved core pathways (Table 2 and S3, Fig. 5). For example, we observed overinduction of specific ROS defense enzymes (catalase, Glutathione S transferase E1, and peroxiredoxin 2450-1) in the *Drosophila G9a* mutant OS response [23]. In the *Drosophila G9a* mutant response to infection, we observed an upregulation of specific antibacterial peptides, CecB, CecC, and AttD [24]. *G9a* knockout MEF cells exposed to interferon show specific overinduction of Toll-like receptors 2 and 4, their intracellular adaptors MYD88 and LCK [25], as well as STX8, a protein involved in lytic granule trafficking that may be required to engulf pathogens after infection [26] and during cytotoxic T-cell responses [27]. G9a knockout ES cells showed overinduction of HIGD1A, a HIF1 induced factor that promotes cell survival during hypoxia by inhibiting the electron transport chain and reducing oxygen consumption [28]. MCF7 cells during hypoxia have overinduced ENDOG, a nuclease that can induce cell death in hypoxia-damaged cells [29].

Taken together, G9a appears to attenuate stress-induced transcription of genes encoding different components involved at all levels of defense, from initial sensing of the stressor and activation of pleiotropic core signaling pathways, down to the executing, stress-tailored resistance mechanisms.

### G9a promotes stress tolerance by maintaining transcriptional homeostasis of metabolic genes

Metabolic reprogramming, including a shift from oxidative phosphorylation to aerobic glycolysis, known as the Warburg effect, is required to fuel an immediate and effective immune response. However, while beneficial at first, this metabolic shift can eventually damage the host as energy reserves are directed away from other essential processes. Consequently, it needs to be under tight control; a prolonged or overactive immune response can lead to death resulting from energy wasting [30]. We showed previously that energy wasting was the cause of death in *Drosophila* G9a mutants exposed to OS [17]. Therefore, tolerance to biotic and abiotic stressors is mediated, at least in part, by limiting energy wasting. In perfect agreement, we observed wide dysregulation of metabolic genes in G9a-depleted conditions across the different stressors and species that we analyzed in this study.

Our analysis provides some insight into the conserved metabolic pathways that are dysregulated in response to G9a deficiency upon exposure to various stressors. Interestingly, genes encoding components of the cell’s major ATP-producing pathways tend to be overrepressed in G9a-depleted conditions. This includes several components of the electron transport chain, key enzymes of mitochondrial and peroxisomal fatty acid oxidation, and key enzymes of glycolysis (Fig. 7). Thus, the capacity for ATP production during stress appears to be compromised in G9a-deficient conditions. In our previous in vivo work examining the response to OS in G9a mutant flies, we observed signs of aerobic glycolysis, including rapid glucose decline and, on the transcriptional level, highly upregulated lactate dehydrogenase [17]. It was thus surprising to observe transcriptional downregulation of glycolysis genes in G9a deficient conditions. Given the high glucose demand that we and others have observed under stress conditions [6, 17, 31], we expected glycolysis to be increased. It is possible, however, that glucose is being used, not for energy production, but for biosynthesis. Glycolysis can be divided into two parts. The first part is unidirectional and consumes 2 ATP molecules per glucose molecule; glucose is converted to fructose 1,6,-bisphosphate. The second part is reversible and generates 4 ATP molecules for every glucose. This energy-generating branch of glycolysis involves seven enzymes that convert fructose 1,6,-bisphosphate to pyruvate and four of these seven enzymes show reduced expression in G9a-depleted conditions. Glycolytic intermediates upstream of these enzymes are essential for de novo synthesis of nucleotides, amino acids, and lipids [9]. It is thus possible that G9a mutants shift the use of glucose towards biosynthesis of nucleotides and amino acids that are required to support their exaggerated transcriptional response to stress. G9a’s role in attenuating stress-induced gene expression may, therefore, promote stress tolerance by allowing for greater glycolytic energy production in place of glycolytic biosynthesis.

One limitation of the three mammalian datasets analyzed in this study is that the data have been acquired from cellular systems that do not reflect the complex local, versus systemic, metabolic reprogramming that occurs in vivo during infection, hypoxia, and likely also other types of stresses. In particular, based on the intrinsic property of cultured cell lines to proliferate, there might be a bias towards glycolytic biosynthesis rather than energy production. An additional limitation is that all of the utilized studies and datasets have, with one or two time points, very limited temporal resolution. It is not guaranteed that the available data reflect comparable phases during the respective stress responses. We therefore expect a considerable amount of false negative genes among the shared group of genes that are dysregulated in G9a-deficient systems, while the uniquely dysregulated genes are for this reason probably overestimated. Ultimately, the net consequences of the integrated transcriptional responses that we describe in this study need to be revealed by targeted in vivo experiments, ideally with spatiotemporal resolution.

### G9a and stress tolerance in human disease

The G9a protein family plays a dual, seemingly paradoxical, function in the context of human disease. Its loss in tumors prevents cancer progression, however, germline mutations in its close paralog and binding partner EHMT1 cause the severe neurodevelopmental disorder Kleefstra syndrome [32]. This paradox may be explained by viewing the G9a family in the context of its arising role in metabolic adaptations to stress. Our previous work suggested that energy availability is essential for stress tolerance [15, 17]. Stress-responsive tissues undergo temporary local and systemic metabolic remodeling in order to cope with the high energy demand of the stress defense [9]. G9a/EHMT acts to limit the amplitude of this response to the minimal yet required level, thereby safeguarding energy resources and minimizing the potentially damaging effect of this shift. This means that G9a/EHMT proteins provide a benefit to tumor cells, which need to maintain proliferation under hypoxia [33]. Tumor cells typically switch to Warburg metabolism with reduced oxidative phosphorylation and increased aerobic glycolysis as a means of energy production. Interestingly, many other cell types undergo similar metabolic remodeling to support their normal biological function. For example, aerobic glycolysis provides energy and biosynthetic intermediates during stem cell differentiation [34], in the neurogenesis of specific fast-expanding neuronal populations [6], and in stimulated neurons that form memory circuits in the brain [35]. It is possible that G9a-mediated stress tolerance also plays a role in coping with these normal metabolic adaptations, potentially explaining developmental and cognitive problems in Kleefstra syndrome, caused by loss of the G9a ortholog EHMT1. In addition, it is emerging that individuals with Kleefstra syndrome suffer from severe regressive periods of unknown trigger and origin [36]. Given our work, it is plausible that stress-induced metabolic dysregulation in these patients, resulting, for example, from infections or other types of stress, can lead to systemic glucose depletion, subsequently affecting brain glucose levels, triggering developmental regression and cognitive decline in analogy to lipopolysaccharide-induced mouse models of delirium and sepsis [37, 38]. Indeed, metabolic reprogramming is of critical importance in sepsis; the process in which an overactivated immune response to infection can cause a fatal outcome referred to as immunoparalysis [11, 39]. G9a/EHMT proteins may be physiological regulators of sepsis or may prove useful to counteract immunoparalysis.

## Conclusions

Taken together, our work suggests the evolutionarily conserved G9a family of epigenetic writers as key master regulators of controlled, appropriately scaled stress responses. These critical responses promote metabolic homeostasis and tolerance to a multitude of biotic and abiotic stressors and, potentially, to demanding physiological conditions.

## Methods

### Differential expression analysis

For the *Drosophila* oxidative stress and *Drosophila* virus RNA sequencing data, fastq files were obtained from the GEO Database (GSE110240, GSE56013) and aligned to *Drosophila melanogaster* reference genome (dmel 6.19) [40] with STAR aligner (version 2.6.0b) [41]. Counts per gene were obtained using htseqcount (version 0.9.1) [42]. For the *Drosophila* OS dataset, we used DESeq2 (version 1.38.0) [43] to normalize count data by library size and calculate differentially expressed genes. Since there were no biological replicates for the virus *Drosophila* dataset, we identified differentially expressed genes with NOISeq (version 2.28.0) [44] using the simulated replicates option (NOIsim) with cutoffs of ≥ 1.2-fold change and a *q*-value of > 0.90. For microarray data, raw files for the interferon MEF, hypoxia ES, and hypoxia MCF-7 datasets were obtained from the GEO database (GSE24776, GSE35061, GSE89891) using the GEO2R package [45]. We used the limma R package (version 3.42.3) [46] to normalize samples using the quantile normalization method and to calculate differential expression with the linear model (lmFit function) and empirical Bayes model (eBayes function). For all datasets, we performed differential expression analysis between the stress-induced samples and the non-induced samples to identify stress-responsive genes with a statistical threshold of > 1.2-fold change and *p*-adj < 0.05. Stress-responsive genes were defined as any gene that passed this threshold in at least one stress-induced timepoint and in either G9a-depleted conditions or controls. Among the stress-responsive genes we identified genes that showed significantly different expression (fold change > 1.2, *p*-adj < 0.05) between G9a-depleted systems and controls at stress-induced time points. Therefore, G9a-dependent stress-responsive genes result from filttering for the intersection of genes that are significantly changed during stress and are significantly different between G9a-depleted conditions and controls.

### Gene ontology analysis

We used g:Profiler (version e94_eg41_p11_9f195a1) [47], to identify enriched GO terms with a Bonferroni-corrected *p* value < 0.05. Enrichment scores for GO terms were calculated as followed: (a/b)/[(c − a)/(d − b)], whereby a is the number of genes associated with the GO term, b is the total number of genes in the principal group, c is the total number of genes in the genome associated with that GO term and d is the total number of genes in the dataset tested for differential expression. We identified GO terms that were enriched in overinduced and overrepressed groups across at least four of five datasets. Enrichment *p* values for each term were calculated using false discovery rate (Benjamini-Hochberg) and visualized using ComplexHeatmap (version 2.7.6.1004) [48].

### Orthology prediction

We used the DRSC Integrative Ortholog Prediction Tool (version 8.0) [49] to identify *Drosophila* orthologs of mouse and human genes. Per gene, we selected the top-scoring gene orthologue and also allowed paralogs with DIOPT scores ≥ 3.

### Gene overlap of stress-specific genes and common stress response genes

Gene overlap was visualized using the R package GeneOverlap (version 1.24.0) [50]. Color code represents the Jaccard coefficient, defined by the size of the intersection divided by the union of the sample sets [51]. *p* value was calculated using false discovery rate (Benjamini-Hochberg). For the total number of genes, we only considered genes with a fly ortholog, according to the above criterion. Enrichment was calculated as described above (section: gene ontology analysis). Percentage of overlap between two datasets was calculated by dividing the number of overlapping genes by the total number of genes in the smaller dataset. Venn diagrams were generated using InteractiVenn [52]. For the non-overlapping genes that were unique to a single dataset, we used g.profiler to identify genes annotated with specific GO terms indicated in Table 2. These genes were then manually annotated based on their specific role in stress response: detection (molecules that sense stress), resistance (factors that directly eliminate the stress), gene regulation (transcription/translation factors and cofactors), signaling (components of signaling pathways), and unknown (annotated based on a mutant phenotype or transcriptional response without mechanistic understanding).

### Protein interaction networks

We constructed a protein interaction network of highly overlapping genes using the STRING app (version 1.5.0) in Cytoscape (version 3.8.0) [53]. For the construction of the networks shown in Figs. 6 and 7, we excluded text-mining as evidence and used a high interaction confidence cut-off of 0.9. The significance of the degree of connectivity was calculated using the String algorithm Random Graph with Given Degree Sequence [22]. KEGG enrichment on genes in the networks was calculated using g:Profiler [47].

## Supplementary Information

**Additional file 1: Table S1.** Differentially expressed genes in the five investigated G9a stressor datasets.

**Additional file 2: Table S2.** Overinduced and overrepressed genes in *G9a* versus control datasets that are shared by three or more studies, two studies, or are uniquely dysregulated.

**Additional file 3: Table S3.** Functional annotation of unique overinduces genes annotated with stress response GO terms.

## Data Availability

Next generation sequencing and microarray datasets analyzed in this study are available in the NCBI GEO repository, under accession numbers: oxidative stress Drosophila (GSE110240) [54], viral infection Drosophila (GSE56013) [55], interferon MEF (GSE24776) [56], hypoxia ES (GSE35061) [57], hypoxia MCF-7 (GSE89891) [58].
